# Physiological and transcriptomic analysis of a yellow leaf mutant in watermelon

**DOI:** 10.1038/s41598-023-36656-6

**Published:** 2023-06-14

**Authors:** Binghua Xu, Chaoyang Zhang, Yan Gu, Rui Cheng, Dayue Huang, Xin Liu, Yudong Sun

**Affiliations:** Huaiyin Institute of Agricultural Sciences of Xuhuai Region in Jiangsu, Jiangsu Academy of Agricultural Sciences, Huai’an, 223001 China

**Keywords:** Biochemistry, Physiology, Plant sciences

## Abstract

Leaf color mutants are important materials for studying chloroplast and photomorphogenesis, and can function as basic germplasms for genetic breeding. In an ethylmethanesulfonate mutagenesis population of watermelon cultivar “703”, a chlorophyll-deficient mutant with yellow leaf (*Yl2*) color was identified. The contents of chlorophyll a, chlorophyll b, and carotenoids in *Yl2* leaves were lower than those in wild-type (WT) leaves. The chloroplast ultrastructure in the leaves revealed that the chloroplasts in *Yl2* were degraded. The numbers of chloroplasts and thylakoids in the *Yl2* mutant were lower, resulting in lower photosynthetic parameters. Transcriptomic analysis identified 1292 differentially expressed genes, including1002 upregulated and 290 downregulated genes. The genes involved in chlorophyll biosynthesis (*HEMA*, *HEMD, CHL1*, *CHLM*, and *CAO*) were significantly downregulated in the *Yl2* mutant, which may explain why chlorophyll pigment content was lower than that in the WT. Chlorophyll metabolism genes such as *PDS*, *ZDS* and *VDE*, were upregulated, which form the xanthophyll cycle and may protect the yellow‒leaves plants from photodamage. Taken together, our findings provide insight into the molecular mechanisms of leading to leaf color formation and chloroplast development in watermelon.

## Introduction

Leaves are crucial organs that produce photosynthates for plant development and growth. Different chlorophyll species and contents determine the color of leaves. A character variation that is easy to recognize in higher plants and occurs with a high frequency is mutation in leaf color. Leaf color mutations are generally expressed at the seedling stage and can be divided into the following types: albino, greenish‒white, white emerald, light green, greenish‒yellow, etiolation, yellow‒green, and striped^1^. Leaf color mutation is formed due to silencing or inactivation of genes controlling chlorophyll biosynthesis and chloroplast development, which directly or indirectly affects chlorophyll synthesis and degradation, ultimately leading to leaf color variation^2^. Therefore, leaf color mutants are ideal materials to study the mechanisms of plant photosynthesis, chloroplast ultrastructure, the chlorophyll biosynthetic pathway, and the expression and regulation of related genes^3^. To date, leaf color mutants have been obtained for various crops, including wheat, carrot, maize, cotton, rice, soybean, and cucumber^4–11^, and used to reveal the mechanisms behind the regulation of chloroplast development and function, chlorophyll biosynthesis, and photosynthesis.

Many yellow‒leaf mutant plants have significantly decreased photosynthetic pigment content and deficient chloroplasts, such as yellow‒green‒leaf 14 (*ygl14*) and 219 (*ygl219*) mutants of rice, yellow‒green leaf wucai (*Brassica campestris* L.) mutant^12–15^. Because a series of enzymatic steps are involved in chlorophyll biosynthesis, blocking any step of chlorophyll synthesis in plants leads to low chlorophyll content, resulting in green‒deficient leaf color^16^. Over 700 sites are involved in leaf color mutations in higher plants, and these sites are involved in the development, metabolism or signal transduction of leaf color formation^1^. The mutant genes that regulate leaf color formation and chloroplast development have been identified in numerous plants^4–15^, such as *OsChlH*, *OsChlI*, and *OsChlD*, which encode the follwing subunits of magnesium chelatase. *YGL1*, which encodes chlorophyll synthase, *OsCAO1* and *OsCAO2*, which encode chlorophyllide a *oxygenases*, *OsDVR*, which encodes an α 8-vinyl reductase that catalyzes the transformation of divinyl Chl a to monovinyl Chl a, and *OsPORA* and *OsPORB*, which encode NADPH: protochlorophyllide oxidoreductase^12,17^. The synthesis of 5-aminolevulinic acid (ALA) is the rate-limiting step for the formation of all plant tetrapyrroles, including chlorophyll and heme. Glutamyl-tRNA reductase is the first committed enzyme in this pathway and is encoded by a small family of nuclear HEMA genes, in which *HEMA1*, *HEMA2*, and *HEMA3* were also identified to be related to leaf color variation^1,13,17^. The proteins encoded by these genes are located in the chloroplast, chloroplast membrane, chloroplast thylakoid membrane, and chloroplast matrix and are mainly involved in chlorophyll biosynthesis and redox-related enzyme regulation^1,18^.

Watermelon [*Citrullus lanatus* (Thunb.) Matsum. et Nakai] is an important vegetable crop worldwide, but thus far, only 5 leaf color mutants have been documented including four spontaneous chlorophyll-deficient mutants^19–23^. Delayed‒green (*dg*) mutants show pale‒green cotyledons and new leaves that turn green as development progresses, which were found from a breeding line Pale90^19,20^. In the variety ‘Sun, Moon and Stars’, spotted mutations are characterized by yellow spots on cotyledons, leaves and fruits^13^. Yellow leaves with reduced chlorophyll content produced by an incompletely dominant allele (*Yl)*^21^. Seedling leaf variegation (*slv*) is a partial chlorophyll deficiency characterized by cotyledons that appear whitish-green, whereas the first leaves display a mosaic-like variegation consisting of scattered white flecks and patches, which are activated by low temperatures in the cultivar New Hampshire Midget (NHM)^22^. A virescent mutant spontaneous originating in ‘Dixielee’, presents young leaves, shoot tips, tendrils and flowers on the main shoot of the juvenile albino mutant, which are all albino during early spring, and the interior portions of leaves gradually become green, while the margins remain albino^23^.

In this study, a novel yellow leaf (*Yl2*) watermelon mutant was identified in an ethyl methanesulfonate (EMS) ‒ mutagenized watermelon population. The mutant exhibited yellow leaves throughout its lifecycle accompanied by decreased chlorophyll content accumulation and impaired chloroplast structure. RNA-seq was used to compare gene expression differences between the *Yl2* mutant and wild-type (WT) to obtain different expression factors, which provides molecular biological evidence for the mechanism of the inconsistent yellow leaf phenotype.

## Results

### Phenotype of the yellow leaf mutant

**Figure 1:**
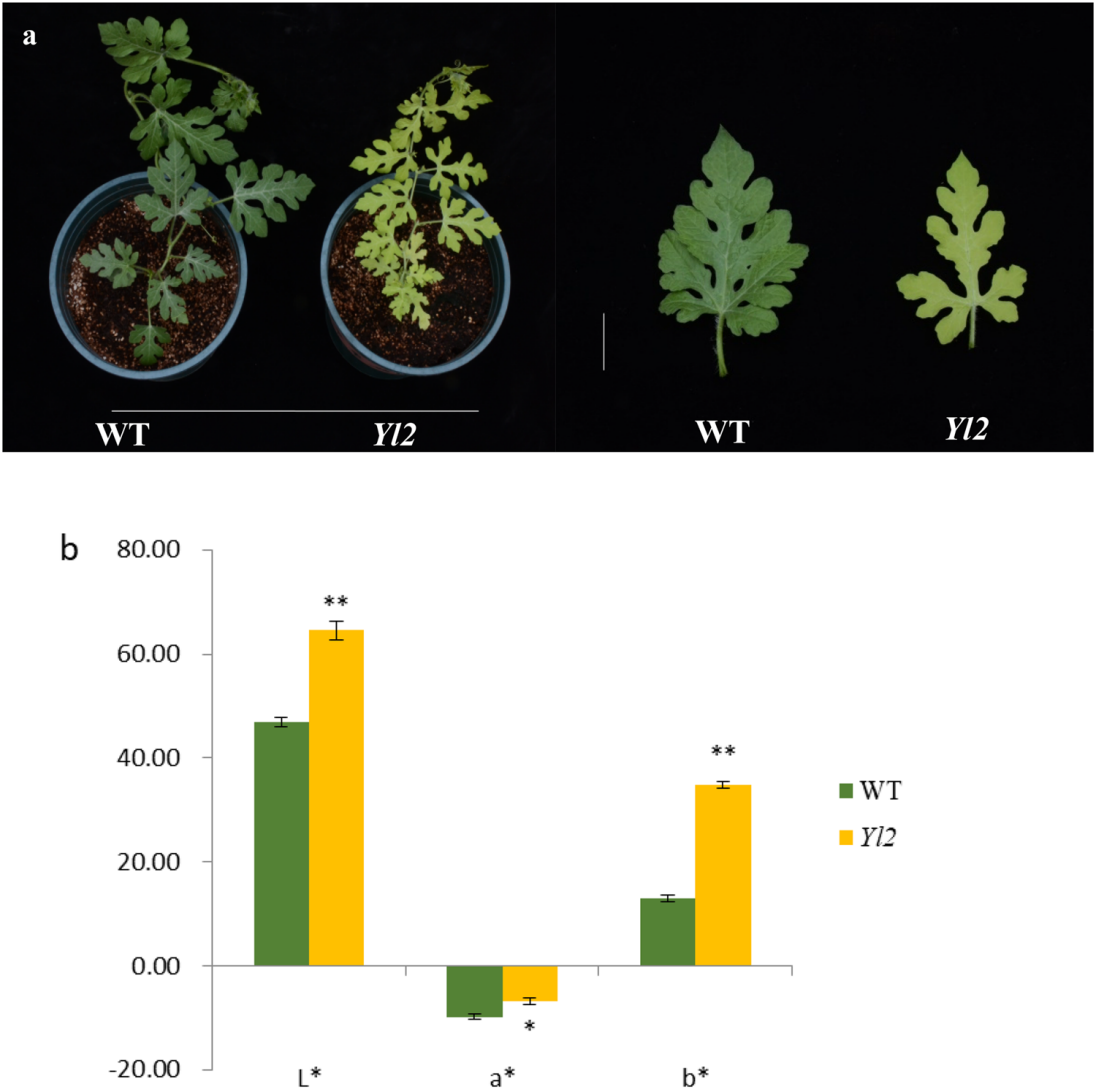
Phenotype of the WT and yellow-leaf mutant (*Yl2*), and their color indices. (**a**) Phenotype of the WT and yellow-leaf mutant (*Yl2*); (**b**) Color indices. The values represent the means ± SDs, and asterisks indicate the statistical significance levels according to Tukey’s test (*P < 0.05, ** *P* < 0.01).

A mutant with yellow leaves was identified in a generation from EMS-mutagenized plants. Compared with the WT, the *Yl2* mutant displayed yellow color throughout its life (Fig. 1). However, the *Yl2* plants showed relatively weaker growth with a much shorter vine length and smaller foliage. After self-pollination, the following types of phenotype segregation were observed in the progeny of the mutant: normal green leaf, yellow leaf, and albino lethal type. The albino plants died when they were seedlings. The yellow leaves of the progeny continue to self‒pollinate, and the three types of phenotype segregation were observed after sowing. The yellow leaf phenotype is likely a heterozygous individual and results form an incomplete dominant allele.

The *Yl2* mutant studied in this work exhibited yellow color throughout life and could normally blossom. Their color indices were measured with two methods. First, the colors were defined according to the Royal Horticultural Society Color Chart (RHSCC). The color of WT was 137B, while that of the *Yl2* mutant was 144A. Second, the colors were expressed as L*, a* and b*. In uniform color space, L* represents lightness, a* represents the ratio of red/magenta and green, and b* represents the ratio of yellow and blue^17^. Compared with those of the WT, the L*, a* and b* values of the *Yl2* mutant were all significantly increased (Fig. 1). These color indices showed that the colors of the WT and *Yl2* mutant were green and yellow, respectively, which was consistent with the visual results.

### Changes in photosynthetic pigments and chloroplast ultrastructure

**Figure 2:**
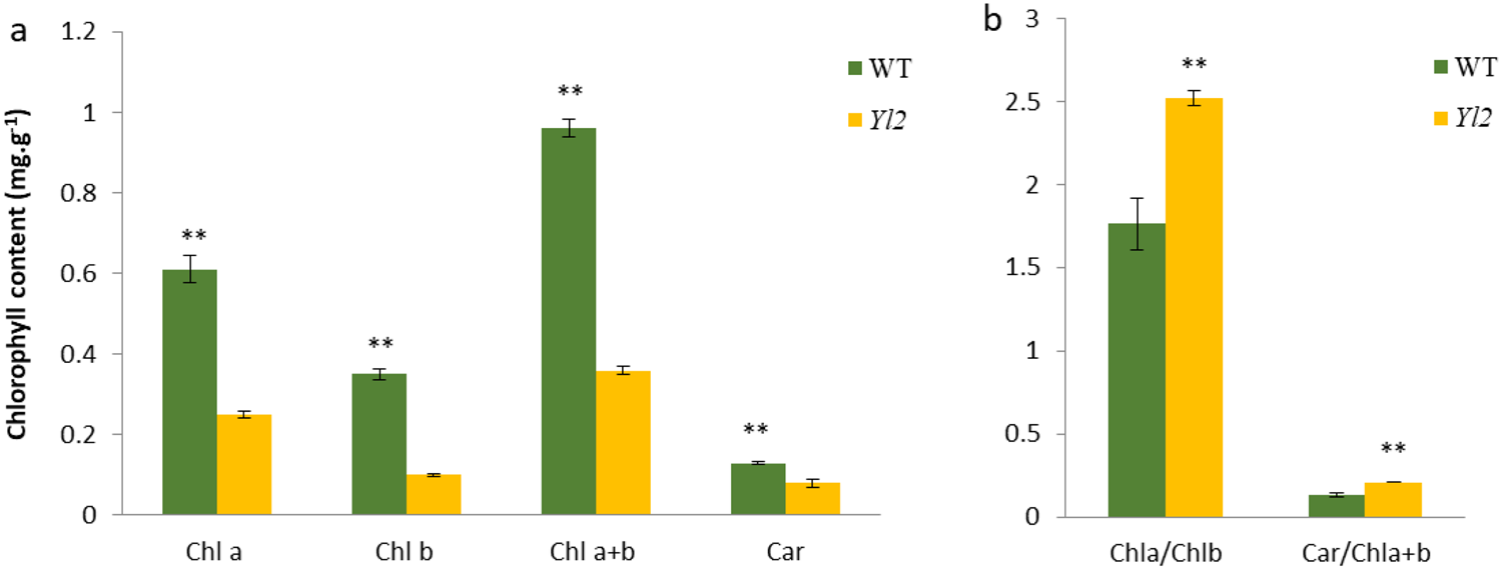
Chlorophyll contents of the WT and yellow-leaf mutant (*Yl2*). The values represent the means ± SDs. (**a**) Contents of Chl a, Chl b, Chl a + b, and carotenoids. (**b**) The ratios of Chl a/Chl b, and Car/Chl a + b. Asterisks indicate the statistical significance levels according to Tukey’s test (*P < 0.05, ** *P* < 0.01).

To investigate the changes in chlorophyll in the yellow‒leaf mutants, we compared the content of some key photosynthetic pigments between yellow leaves and WT plants. The Chl a, Chl b, Chl a+b and carotenoid contents differed between the WT and *Yl2* mutant (Fig. 2a). The contents of Chl a, Chl b, Chl a+b and Car in the *Yl2* mutant were 0.25 mg/g, 0.10 mg/g, 0.35 mg/g and 0.08 mg/g which were significantly reduced by 58.32%, 70.95% , 62.90%, and 51.06%, respectively, compared with those in the WT. The ratios of Chl a/Chl b and Car/Chl a+b in the *Yl2* mutant were 2.53 and 0.21, respectively, which were significantly higher than those in the WT (Fig. 2b). These data indicated that the yellow leaves of the *Yl2* mutant were related to the decreased chlorophyll content, especially chlorophyll b.

**Figure 3:**
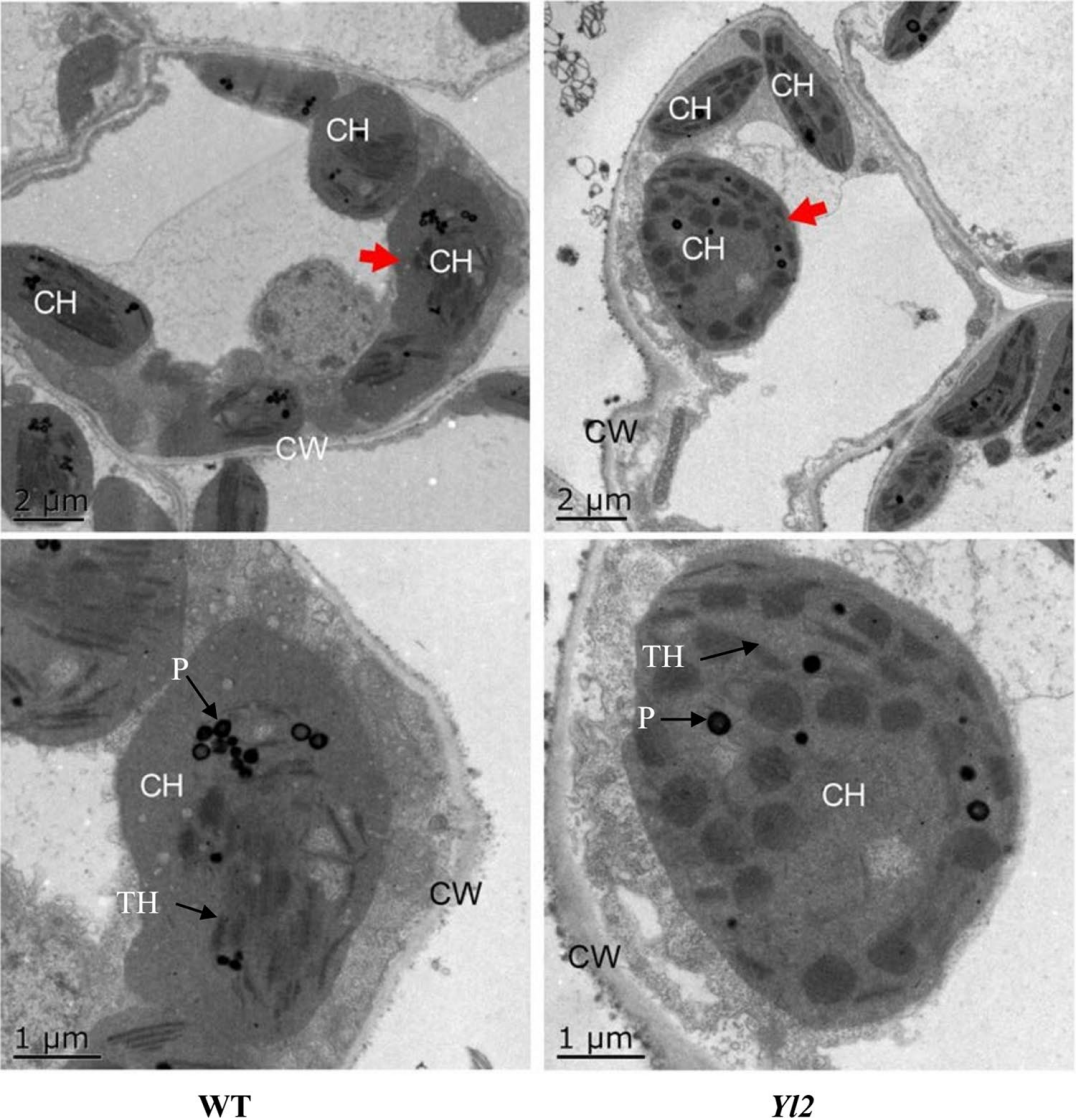
Cellular ultrastructure of the WT and yellow-leaf mutant (*Yl2*). The lower scanning electron micrograph is a partial enlargement of the upper micrograph marked by an arrow. CW, cell wall; CH, chloroplast; TH, thylakoid; P, plastoglobulus.

To further detect the difference between the *Yl2* mutant and WT, ultrastructural analysis of chloroplasts was performed. More chloroplasts were found in WT leaves than in the *Yl2* mutant leaves (Fig. 3). Significant differences were also observed in the arrangement and number of thylakoids in WT and mutants. The thylakoids in WT were neatly arranged, concentrated, and numerous, while the thylakoids in the *Yl2* mutant were smaller, less abundant, thinner, and disordered (Fig. 3). Thus, the mutation in *Yl2* affected chloroplast development.

### Photosynthetic characterization and fluorescence kinetic parameter assessment

**Figure 4:**
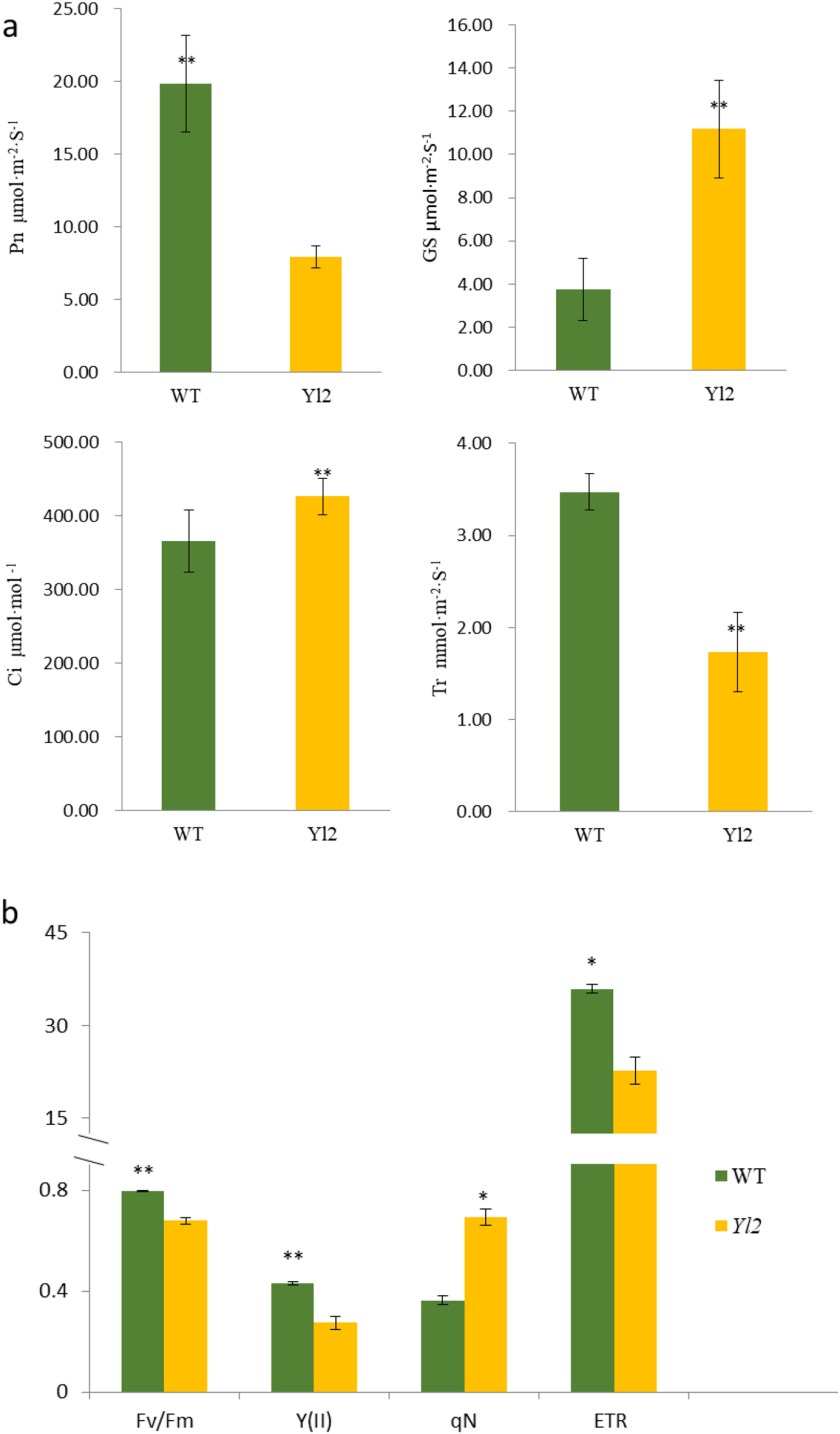
Photosynthetic characteristics and chlorophyll fluorescence parameters of the WT and yellow-leaf (*Yl2*) mutant. The values represent the means ± SDs. Asterisks indicate the statistical significance levels according to Tukey’s test (*P < 0.05, ** P < 0.01). (**a**) Photosynthetic characteristics. (**b**) Chlorophyll fluorescence parameters.

To investigate whether the reduction in chlorophyll pigment content and chloroplast development defects affected photosynthesis in *Yl2* mutants, we examined photosynthesis‒related parameters and chlorophyll fluorescence. The net photosynthetic rate (P_n_) and transpiration rate (Tr) were significantly decreased by approximately 60% and 50%, respectively, in the *Yl2* mutant compared with the WT (Fig. 4a). The stomatal conductance (Gs) and intercellular CO_2_ concentration (Ci) of *Yl2* were significantly higher than those of WT (Fig. 4a). Thus, the photosynthetic characteristics of *Yl2* were negatively impacted.

Fluorescence kinetic parameters reflect the ability of plants to absorb and transfer light. To analyze the relationship between their leaf color changes and chlorophyll fluorescence parameters, relative parameters were investigated to evaluate light transfer efficiency. The effective quantum yield of photochemistry of PSII (Fv/Fm) and actual photochemical quantum yield (Y(II)) in the *Yl2* mutant were significantly lower than those in the WT, suggesting significant photo‒inhibition in the *Yl2* mutant^1^. The electron transport rate (ETR) of *Yl2* was decreased by 36.91% compared with the WT, suggesting that the *Yl2* mutant was less efficient in electron transfer, and utilization was lower than that of the WT. The nonphotochemical quenching (qN, percentage of PSII efficiency loss due to heat dissipation) was increased by 81.97% in the *Yl2* mutant (Fig. 4b). These results indicate that the reduction in chlorophyll and carotenoid content might account for the low photosynthetic rate and light-use efficiency in the *Yl2* mutant.

### Transcriptomic analysis

**Figure 5:**
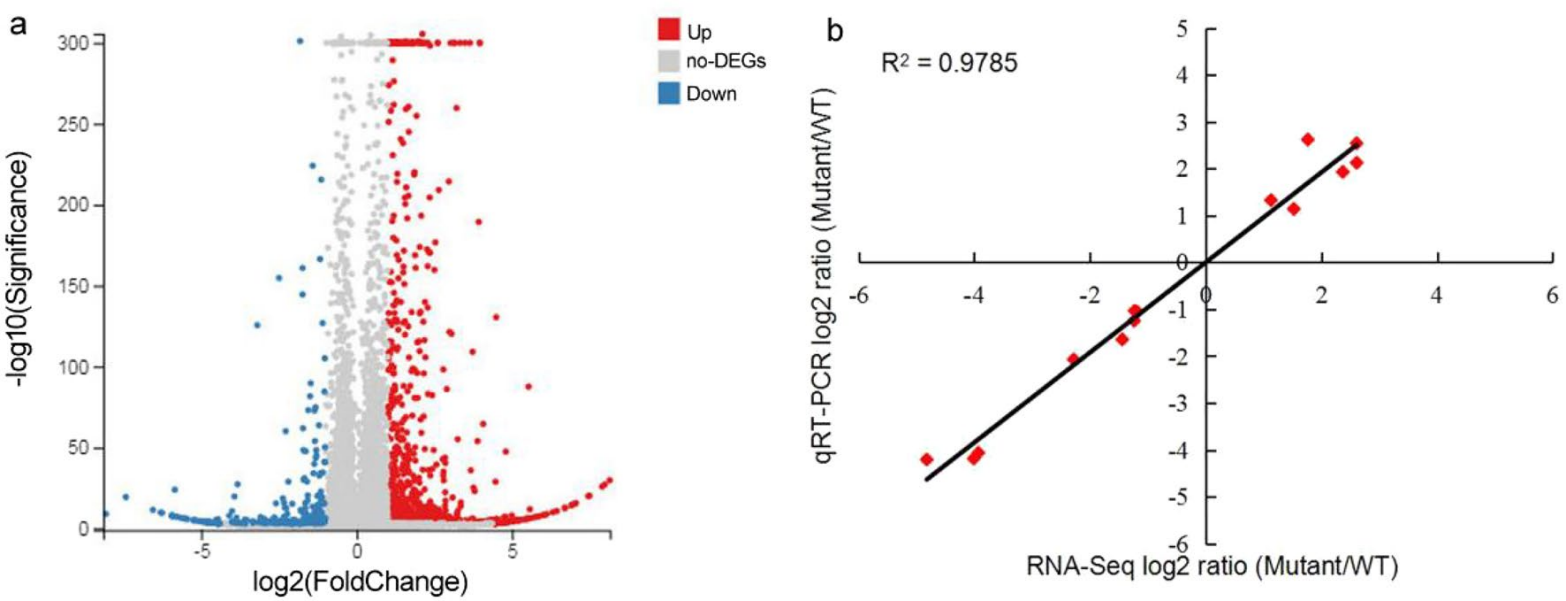
RNA-Seq analysis of WT and yellow-leaf mutant. (**a**) Volcano plot of differentially expressed genes (DEGs). The X axis represents the log2-transformed fold change, the Y axis represents the − log10-transformed significance, the red points represent upregulated DEGs, the blue points represent downregulated DEGs and the gray points represent non-DEGs. (**b**) Correlation of RNA-Seq (X-axis) and qRT‒PCR data (Y-axis). Correlation analysis was carried out for 14 DEGs with log2 ratios ≥ 1.00 or ≤ 1.00.

To explore the molecular mechanism of the yellow‒leaf phenotype of the *Yl2* mutant, leaf transcriptomes from sequencing libraries of WT and *Yl2* individuals were assessed, 79.12 and 78.05 M total raw reads in WT and *Yl2*, respectively. After removing low‒quality sequences, filtering adapters and ambiguous reads, we obtained approximately 67.30 and 67.77 M total clean reads in the WT and *Yl2*, respectively. Then, these total clean reads were mapped to the watermelon genomic database, with match ratios of 85.21% and 84.73%, respectively (Table S1). Differential gene expression analysis was conducted between the WT and *Yl2* mutant. A total of 1292 DEGs (*p* value < 0.001, |fold-change| > 2) were detected, including 1002 up regulated DEGs and 290 downregulated DEGs (Fig. 5a).

**Figure 6:**
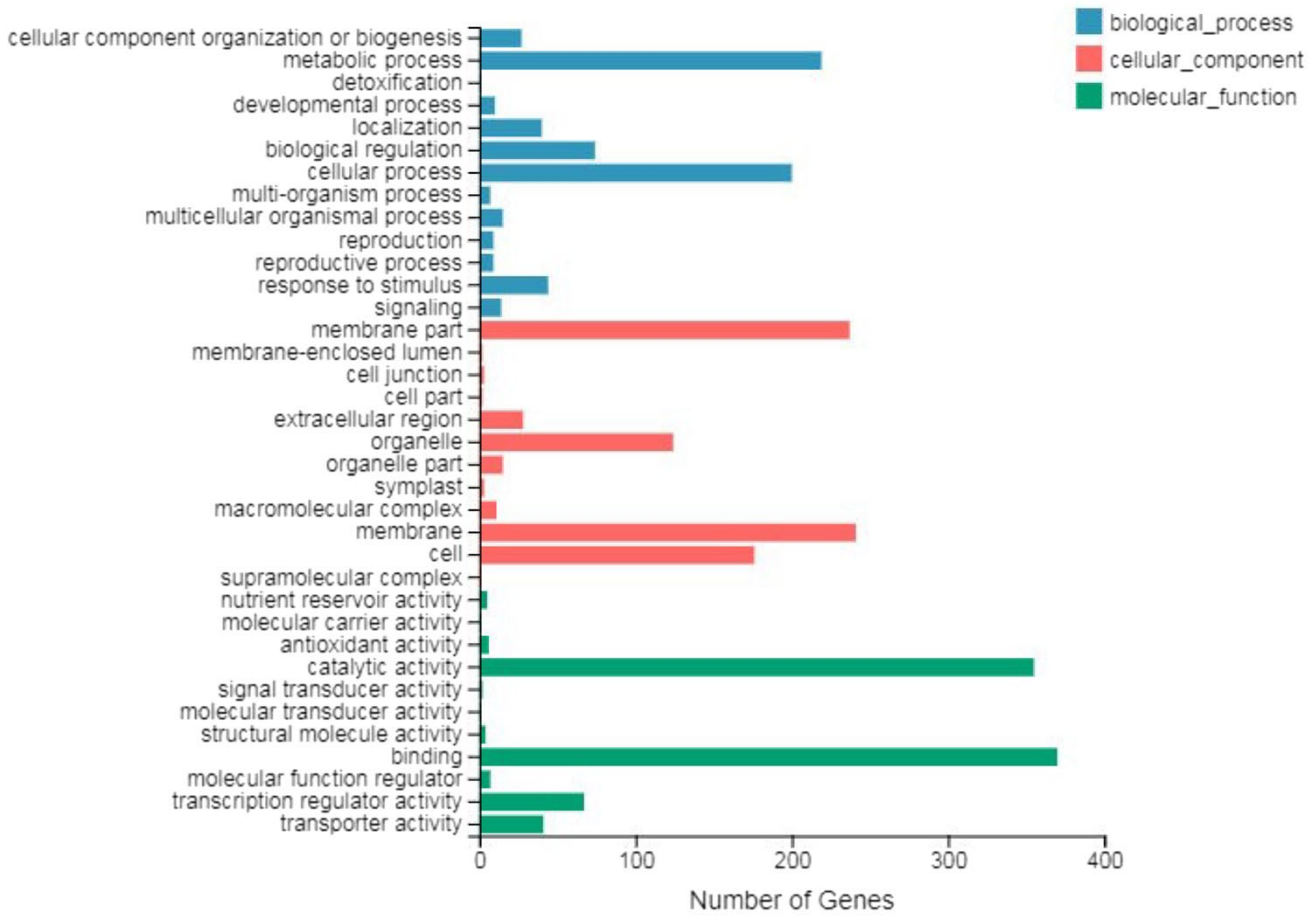
GO classification of DEGs. The X-axis represents the number of differentially expressed genes; the Y-axis represents the GO term.

**Figure 7:**
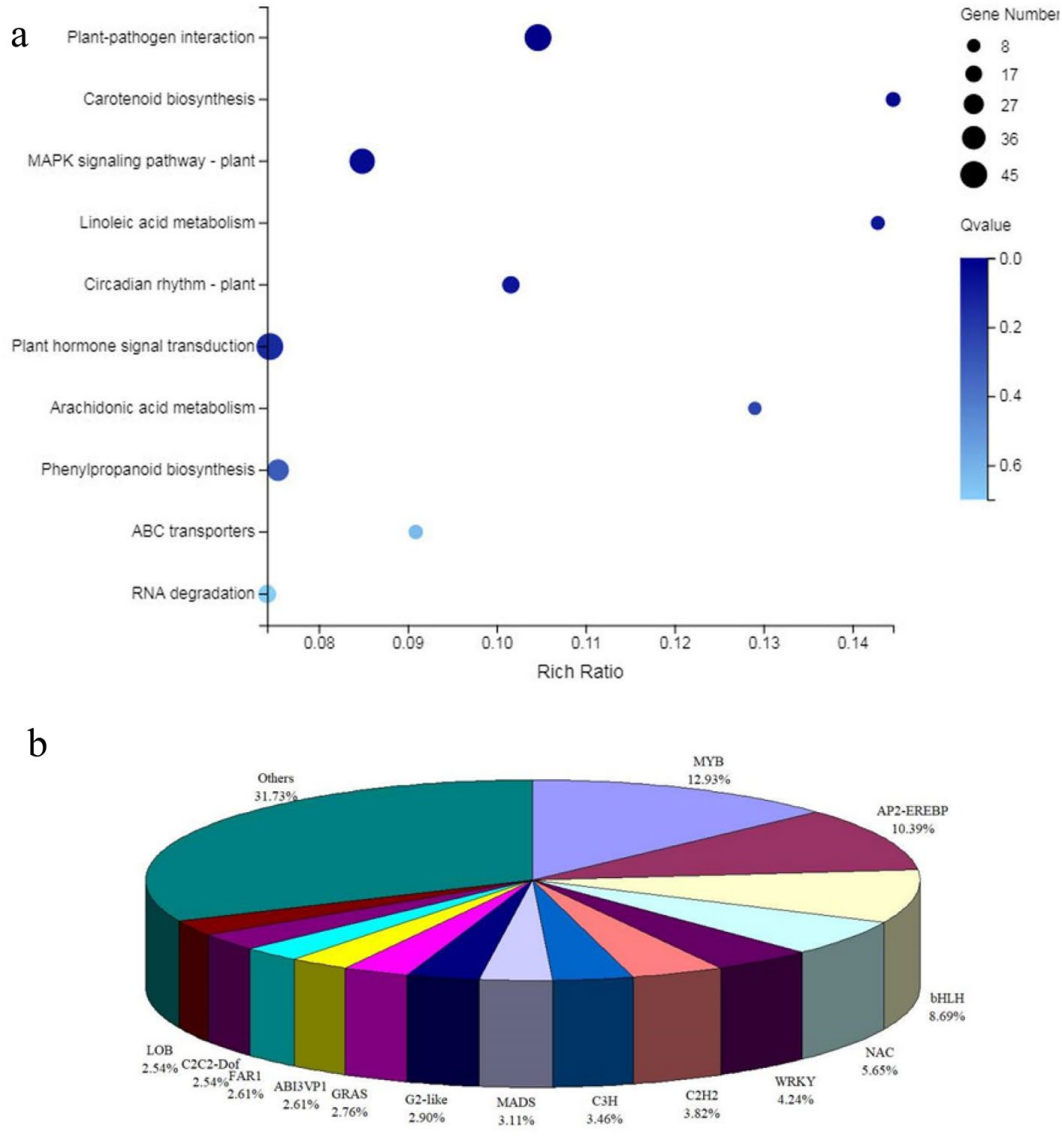
Pathway functional enrichment of DEGs. A: The X-axis represents the enrichment ratio, the Y-axis represents the pathway name, coloring indicates q-value, a lower q-value indicates more significant enrichment, and the point size indicates the DEG number.

To validate these DEGs, the expression levels of 14 randomly selected genes were analyzed by qRT‒PCR, by comparison, a significant positive correlation (*R*^2^ = 0.9785) was found between the RNA-Seq data and qRT‒PCR results. Moreover, the regression slope for RNA-Seq versus qRT‒PCR was close to 1.0, suggesting that the RNA-Seq data were credible (Fig. 5b). The functions of these DEGs were classified according to the GO database using the Blast2GO software suite (Fig. 6). These DEGs were involved in biological processes (669), cellular components (843) and molecular function (859). The more abundant biological processes were mostly in the metabolic process (219) and cellular process (200); the more significant cellular components were the membrane (241) and membrane part (237); and the more significant molecular functions were binding (370) and catalytic activity (355). In addition, all DEGs were matched and assigned to 113 KEGG pathways, and only 3 pathways, including plant‒pathogen interaction, MAPK signaling pathway-plant and carotenoid biosynthesis, achieved a Q-value ≤ 0.05 (Fig. 7a).

Furthermore, transcription factors are key regulatory proteins that play important roles in regulating gene expression in various plant biological processes. Putative transcription factors associated with leaf coloration were analyzed. A tolal of 1415 genes were putatively identified as transcription factors, and they were associated with 59 transcription factor families. Of them, the most abundant transcription factor family was the MYB superfamily (183, 12.93%), followed by AP2-EREBP (147, 10.39%), bHLH (123, 8.69%), and NAC (80, 5.65%) (Fig. 7b).

### Identification of differentially expressed genes (DEGs) associated with leaf coloration

**Figure 8:**
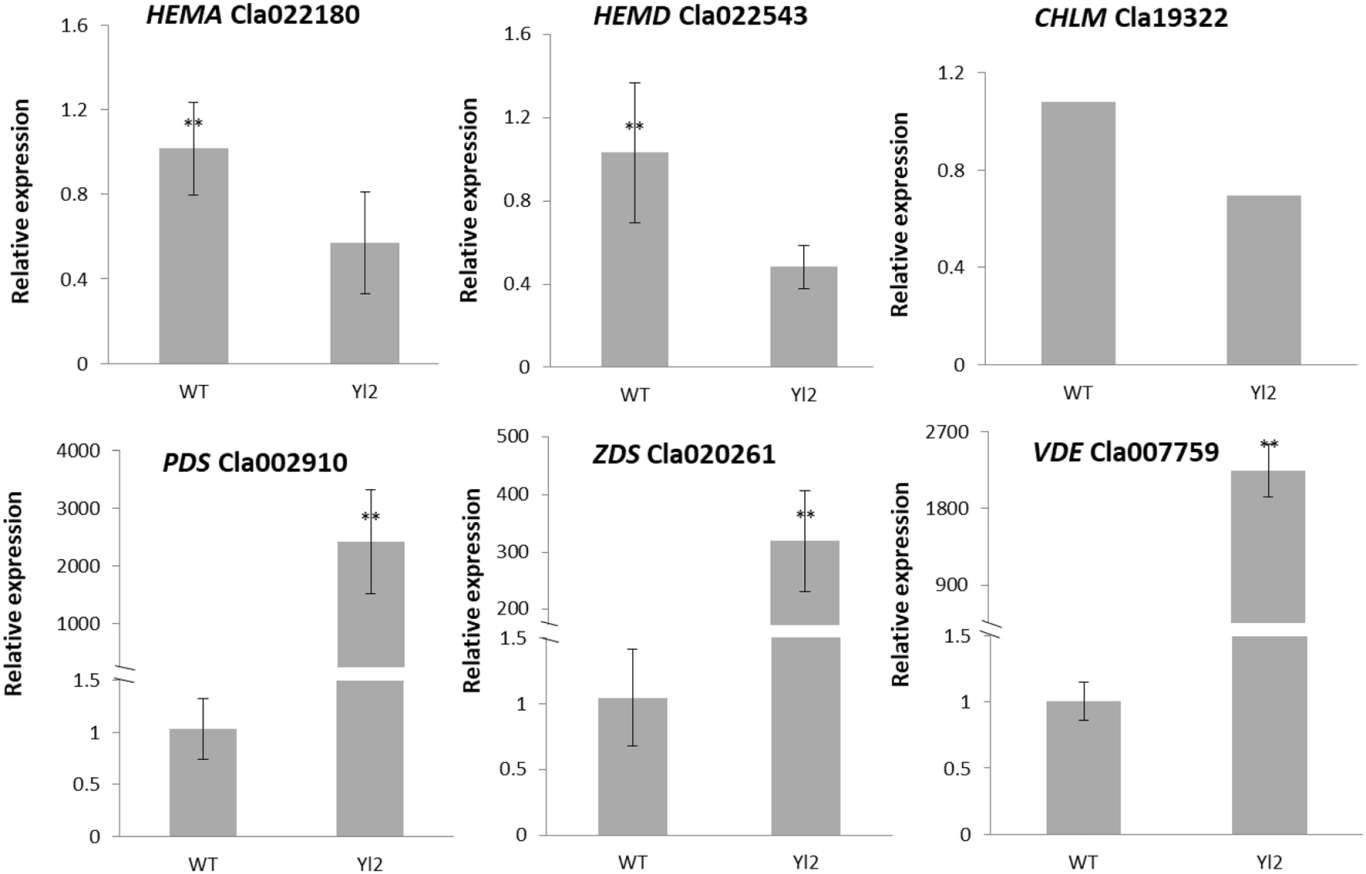
Expression profiles of genes involved in chlorophyll metabolism in the WT and yellow-leaf mutant (*Yl2*). The values represent the means ± SDs; and asterisks indicate the statistical significance levels according to Tukey’s test (*P < 0.05, ** *P* < 0.01).

To explore the DEGs associated with leaf coloration, the expression profiles of genes involved in chlorophyll biosynthesis in the WT and *Yl2* mutant were analyzed according to their FPKM values. In chlorophyll metabolism, we found 9 genes downregulated, including the glutamyl-tRNA reductase 1 gene (*HEMA*, *Cla022180*, and *Cla012951*), uroporphyrinogen-III synthase gene (*HEMD*, *Cla018703*, and *Cla022543*), protoporphyrinogen oxidase gene (*HEMG*, *Cla006520, Cla005862*), magnesium chelatase subunit H gene (*CHLH*, *Cla002769*), magnesium chelatase gene (*CHL1*, *Cla009670*), magnesium protoporphyrin IX methyltransferase gene (*CHLM*, *Cla019322*), chlorophyll synthase gene (*CHLG*, *Cla018095*), chlorophyllide a oxygenase gene (*CAO*, *Cla013939*), and pheophorbide a oxygenase gene (*PAO*, *Cla021047*). The expression levels of these genes in the *Yl2* mutant were all lower than those in WT (Fig. 8). Moreover, we found that carotenoid biosynthetic genes, including the phytoene synthase gene (*PSY*, *Cla003169*), phytoene desaturase gene (*PDS*, *Cla002910*), zeta-carotene desaturase gene (*ZDS*, *Cla020261*), and the xanthophyll cycle genes including zeaxanthin epoxidase gene (*ZEP, Cla020214*, *Cla015814*) and violaxanthin de-epoxidase gene (*VDE, Cla007759*), were upregulated in the *Yl2* mutant (Fig. 8). These results indicate that the yellow leaves exhibit largely different metabolic activities from those of the WT leaves.

## Discussion

Leaf color mutants are ideal models for investigating photosynthetic pigment metabolism and functional chloroplast development. We developed a yellow-leaf mutation with EMS (Fig. 1), which was controlled by an incomplete dominant mutation of the nuclear gene. The homozygote is albino and dies after cotyledons unfold, while the heterozygote has yellow leaves during the entire growth stage, and can bear fruit and produce seeds.

### The yellow leaf phenotype is closely associated with chlorophyll content and chloroplast ultrastructure

Chlorophyll a, chl b and carotenoids are the main photosynthetic pigments in the leaves of higher plants. The main reason for an abnormal leaf color is an increase or decrease in chlorophyll content in the leaves. In our study, the contents of chlorophyll and carotenoids in the *Yl2* mutant were significantly reduced, directly causing yellow leaves (Fig. 2). The same results have been reported for other crops^11–15^. The decreased pigments in *Yl2* probably affects the chloroplast development and photosynthesis process^11,14,15^.

Chloroplasts are not only the site of photosynthesis but also an important organelle for energy transformation and storage in green plants; therefore, yellow-leaf mutant plants showed slower and weaker growth than that of WT plants. The pigments in plant leaves are mainly arranged in and around photosystems that are embedded in the thylakoid of chloroplasts. The chloroplast structure of the *Yl2* mutant in our study was damaged during chloroplast formation, which resulted in the abnormal development of thylakoids (Fig. 3), eventually leading to decreased chlorophyll content compared to that of the WT. The chloroplasts in many leaf color mutants, such as cucumber and rice were also imperfect, and contained looser thylakoids than those in the WT^11–16^. In the yellow‒leaf mutant (*YX-yl*) of broomcorn millet, chloroplast development was inhibited owing to degeneration of the chloroplast ultrastructure, which exhibited fewer grana thylakoids and almost no stroma thylakoids^14^. A green‒yellow leaf wucai (*Brassica campestris* L.) mutant exhibited fewer chloroplasts per cell and looser stromal lamellae^15^. The chlorophyll content in an irregularly striped rind mutant cucumber peel was lower, and the number and structure of the chloroplasts also changed in the peel cells of the *ist* mutant compared with that of the WT^24^. The decreased chlorophyll contents and poorly developed chloroplasts might result in poor chloroplast function and accelerated leaf yellowing.

### The yellow leaf phenotype is closely related to photosynthesis

The lower chlorophyll pigment content, combined with damaged chloroplasts, might account for the poorer photosynthetic performance and light transfer efficiency in the leaf color mutant^14^. In previous studies, lower chlorophyll content was found to be closely related to the photosynthetic rate and light-use efficiency. The Pn, Gs, and Tr of *ygl14* were significantly lower than those of the WT, however, the intercellular CO_2_ concentration was significantly increased^13^. The photosynthetic parameters in the *ygl54* mutant were all significantly decreased compared to those in the WT^25^. Chlorophyll fluorescence parameters can effectively reflect the absorption, utilization, dissipation and distribution of light energy by plants. The results of photosynthetic performance and fluorescence kinetic parameters revealed that yellow color had a negative impact on the photosynthetic machinery of *Yl2* leaves compared with the WT (Fig. 4)^26^. The results might be due to the different levels of chlorophyll deficiency and chloroplast development inhibition.

### The expression pattern of genes related to chlorophyll biosynthesis and chloroplast development may account for the yellow leaf phenotype

Chlorophyll and carotenoid biosynthesis are determined by complex biological processes, and blocking any step in this process can lead to a decrease in chlorophyll or carotenoid content, which in turn results in a change in leaf color^1,27–30^. Previous results suggested that changes in genes related to chlorophyll metabolism and chloroplast development might result in a yellow‒leaf phenotype. Genes such as *CHLD*, *CHLI*, *CHLM*, and *HEMA* in the yellow‒green leaf *ygl14* mutant were downregulated^13^. *CAO*, *CHLH* and several key synthesis genes in the chlorophyll biosynthesis pathway in the irregularly striped rind mutant (*ist*) cucumber were significantly downregulated, which may directly cause of the rind appearance changes in the mutant^24^. Virus-induced gene silencing of the* CHL1* and *CHLD* (*VIGS*-*CHL1* and *VIGS*-*CHLD)* genes both resulted in a yellow leaf phenotype in pea, and lower chlorophyll accumulation and undeveloped thylakoid^31^. *OsHemA* plays an indispensable role in Chl biosynthesis and even plant growth and development, and its downregulated expression reduces Chls and Cars, which in turn results in light green leaves of rice^32^. *HEMD*, uroporphyrinogen III synthase, cyclizes and isomerizes hydroxymethylbilane to produce uroporphyrinogen III in the plastid. The chlorophyll content in rhizomes of Cymbidium increased after light treatment, and the expression levels of *HEMD* and *CHLG* gradually increased^33^.In our study, the genes involved in this process, such as *HEMA*, *HEMD, and CHLM*, exhibited significantly downregulated expression in the *Yl2* mutant (Fig. 8). This explained why the content of chlorophy pigment in *Yl2* mutant was much lower than that in the WT.

Phytoene desaturase (*PDS*) is a key enzyme in the carotenoid biosynthesis pathway and plays a critical role in the production of carotenoids, xanthophylls, alpha-carotene, and beta-carotene^34,35^. Knocking out the function of the *PDS* gene leads to albinism and slow plant growth^36^. *ZEP* and *VDE*, which are the key genes in the xanthophyll cycle, were upregulated in the *Yl2* mutant leaves. Previous studies have shown that the xanthophyll cycle mechanism protects plants against photodamage^34^. Under strong light, the expression of *VDE* in *Arabidopsis* leaves increased, which was beneficial to the transformation of Viola yellow to anther yellow and zeaxanthin, consumed extra light energy, and protected photosynthetic organs from strong light^37^. In the broomcorn millet yellow leaf mutant, *PmVDE* genes related to chlorophyll metabolism and chloroplast development were upregulated^14^. The analysis of these differentially expressed genes, also provides a theoretical basis for the molecular mechanism of watermelon yellow leaf formation.

These results showed that chlorophyll metabolism, chloroplast development, and chloroplast transcription were impaired in *Yl2* plants, leading to chloroplast thylakoid degradation, reduced chlorophyll content, and yellow-leaves. Our study provides an essential reference for the investigating leaf mutant mechanisms in watermelon.

## Methods

### Plant materials and phenotypic data collection

The *Yl2* mutant was isolated from an M_0_ family derived from an EMS-mutagenized watermelon “703”. “703” was a homozygous inbred line. The *Yl2* mutant was self-pollinated. The following types of phenotype segregation occur in the progeny of the mutant: normal green leaf, yellow leaf and albino lethal type. The albino plants aborted in the seedling stage. The yellow leaves of the progeny continue to self-pollinate, and the three types of phenotype segregation were observed after sowing. Watermelon “703” (WT) and its *Yl2* mutant were used as materials in this study. After the plants were grown for 2 months, samples were taken for RNA-seq and leaf anatomy observation. In addition, photosynthetic characteristics, chlorophyll fluorescence and other physiological indices were measured.

### Color indices and pigment content measurement

The color of the first fully expanded leaves was compared with the RHSCC, and then its surface color was measured with a hand-held RM200QC spectrocolorimeter (X-Rite, Switzerland) using three color parameters including L*, a* and b* values. Chlorophyll and carotenoid contents were all determined according to the methods reported by Chen^38^. The absorbances of Chl a, Chl b, Chl a+b and Car was determined in the spectrophotometer at 663 nm, 645 nm, 652 nm and 440 nm, respectively.

### Photosynthetic characteristics and chlorophyll fluorescence parameter measurement

Photosynthetic parameters were measured with a portable photosynthesis system (Li-Cor LI-6400, USA) on a cloudless day. The net photosynthesis rate (P_n_) and transpiration rate (Tr) were recorded. Moreover, the chlorophyll fluorescence parameters were measured with a chlorophyll fluorescence spectrometer (Heinz Walz GmbH 91090 Effeltrich, Germany). The minimum fluorescence (Fo), maximum fluorescence (Fm), maximum quantum yield of PSII (Fv/Fm=(Fm-Fo)/Fm), the actual photosynthetic efficiency of photosystem II (Y(II) =ΔF/Fm′= (Fm′−F)/Fm′), nonphotochemical quenching (qN), and electron transport rate (ETR) were calculated and recorded^39,40^. Measurements were performed on the third, fully expanded leaf. Ten individuals were measured and repeated twice, and then the average was taken.

### Anatomy observation

The anatomical details of leaves were observed by transmission electron microscopy (Tecnai 12, Philips, Holland). The second young leaves from wild type and *Yl2* mutant were cut into 1× 0.5 cm^2^ sections, and fixed overnight in a solution of 2.5% glutaraldehyde. The fixed leaves were washed 3 times with 0.1 mol/L phosphate buffer for 15 min, and postfixed with 1% osmium tetroxide for 4 h at room temperature (25 °C). Then the leaves were dehydrated using 50%, 70%, 85%, 95% and 100% gradient ethanol for 15 min each. Moreover, they were treated with 100% acetone solution (15 min) and acetone solution containing anhydrous sodium sulfate (15 min), infiltrated in Spurr resin and then hardened at 70 °C for 24 h. Sections (70 nm thick) were cut with a diamond knife using a Leica EM UC6 ultramicrotome (Leica Co., Austria) and stained with 1% uranyl acetate in 70% methanol, and 1% lead citrate before examination.

### RNA-Seq and DEG analysis

Total RNA was extracted from the leaves of the WT and the *Yl2* mutant using a MiniBEST Plant RNA Extraction Kit (TaKaRa, Japan). RNA quality and purity were assessed using 1.0% agarose gels and a NanoDrop 8000 spectrophotometer (Thermo Scientific, Waltham, USA), and the integrity was evaluated by the Agilent 2100 Bioanalyzer (Agilent Technologies, Santa Clara, USA). Six sequencing libraries (WT and *Yl2*, three replicates) were prepared and sequenced by Beijing Genomics Institute Co., Ltd. (Shenzhen, China) using an Illumina HiSeq™ 4000 system (Illumina Inc., San Diego, CA, USA). The watermelon 97103 genome was used as the reference genome for sequence alignment. Raw reads from RNA-seq were filtered to obtain high-quality clean read pairs. TopHat2 software and Cufflinks were used to complete comparison and transcript splicing analysis respectively, and all genes were quantitatively analyzed and calculated. The quantitative index of the expression level of each gene was calculated based on the fragments per kilobase per million mapped reads (FPKM). The differentially expressed genes (DEGs) were screened according to the criteria of *p* value < 0.001 and |log2 (fold change) > 2. DEG functions were explored through GO and KEGG pathway analyses and the terms with *p* values ≤ 0.05 were defined as significantly enriched. These analyses were performed to identify significantly enriched metabolic pathways. The raw reads with available data were deposited in the sequence read archive of the National Center for Biotechnology Information under accession number PRJNA890394.

### RT‒qPCR

Gene transcript levels were analyzed using quantitative real-time PCR (qRT**‒**PCR) with a BIO-RAD CFX ConnectTM Optics Module (Bio-Rad, USA). cDNA was synthesized from RNA using the PrimeScript^®^ RT reagent kit with gDNA Eraser (TaKaRa, Japan). All gene-specific primers in this study (Table S2) synthesized by Shanghai Sangon Biological Engineering Technology & Services Co., Ltd. (Shanghai, China). qRT‒PCR was performed using SYBR® Premix Ex Taq^TM^ (Perfect Real Time) (TaKaRa, Japan) and contained 12.5 µL 2 × SYBR Premix Ex Taq^TM^, 2 µL cDNA solution, 2 µL mix solution of target gene primers and 8.5 µL ddH_2_O in a final volume of 25 µL. The amplification was carried out under the following conditions: 50 °C for 2 min followed by an initial denaturation step at 95 °C for 30 s, 40 cycles at 95 °C for 5 s, 50 °C for 15 s, and 72 °C for 30 s. Relative gene expression levels of target genes were calculated by the 2^−∆∆Ct^ comparative threshold cycle (Ct) method ^38^. The Ct values of the triplicate reactions were gathered using Bio-Rad CFX Manager V1.6.541.1028 software. The watermelon *Actin* gene was used as an internal reference and PCR analysis for each gene was performed with three biological and three technical replicates.

### Statistical analysis

Primers were designed using the Primer 5.0 program. All data are the means of three replicates with standard deviations. The results were analyzed for variance using the SAS/STAT statistical analysis package (version 6.12, SAS Institute, Cary, NC, USA).

### Permit statement

Experimental research and field studies on watermelon, including the collection of plant material, complied with institutional, national, and international guidelines and legislation. Permissions were not required for WT and yellow leaf mutant of watermelon collections, because the WT is a cultivar selected by the authors.

## Supplementary Information

Supplementary Table S1.

Supplementary Table S2.

## Data Availability

The raw reads with available data were deposited in the sequence read archive of the National Center for Biotechnology Information under accession number PRJNA890394.
