# Supplementary material for: Physiological and transcriptomic analysis of a yellow leaf mutant in watermelon
Source: Sci Rep. 2023 Jun 14;13:9647. doi: 10.1038/s41598-023-36656-6 (PMC10267204; doi:10.1038/s41598-023-36656-6)
Supplement: Supplementary file 1 — Supplementary Table S1. [file 41598_2023_36656_MOESM1_ESM.doc]

**Table S1.** Summary of sequencing reads in WT and Mutant.

| **Sample name** | **Total raw read (M)** | **Total clean read (M)** | **Clean reads Q20 (%)** | **Clean Reads Q30 (%)** | **Total mapping (%)** |
| --- | --- | --- | --- | --- | --- |
| WT1 | 78.06 | 67.34 | 98.11 | 94.5 | 84.54 |
| WT2 | 79.65 | 67.38 | 98.41 | 95.27 | 85.17 |
| WT3 | 79.65 | 67.19 | 98.4 | 95.22 | 85.92 |
| Mutant1 | 78.05 | 67.61 | 98.15 | 94.63 | 83.68 |
| Mutant2 | 78.05 | 68.1 | 98.13 | 94.58 | 84.09 |
| Mutant3 | 78.06 | 67.62 | 98.09 | 94.48 | 86.43 |
