# Supplementary material for: Physiological and transcriptomic analysis of a yellow leaf mutant in watermelon
Source: Sci Rep. 2023 Jun 14;13:9647. doi: 10.1038/s41598-023-36656-6 (PMC10267204; doi:10.1038/s41598-023-36656-6)
Supplement: Supplementary file 2 — Supplementary Table S2. [file 41598_2023_36656_MOESM2_ESM.doc]

**Table S2. Primers used for qRT-PCR.**

| **Gene ID** | **Forward primer (5' - 3')** | **Reverse primer (5' - 3')** |
| --- | --- | --- |
| *Cla005404* | TATGGTAGAAACAGAGTTGC | TTCTGCAAATCCTCGGAAC |
| *Cla003169* | AAAGGAGCGACAAAGAGC | CCAGCAAATCATAGGGAC |
| *Cla005457* | ATGTTCGCCTTCTCCGTTCT | GGAAGGCCATTGAGAGGAAG |
| *Cla020673* | ATGGGCGAGATTTTTGTATC | GGCCTATCTTCCAAAATCTG |
| *Cla006026* | AGAGTGATTGAAGCAAGGAG | AGTCCTGCTACCTTTCCCAG |
| *Cla015176* | ATCGTCAACGGCATACAT | CAATCTCACTCCCTTCCA |
| *Cla008356* | ATGAGTTCCAATGGCGTTTC | TTGTCCATAGGCTTTAGCAT |
| *Cla016575* | GCATTGTCTTCGCCACTG | CGGCTTTTTCTACACTTTCA |
| *Cla002772* | TTGAGTCTTTGAAGCCTG | TAGCATCTCCAATGGTGA |
| *Cla018502* | ACAGTGTAGCAGAAAGGAT | TGAAGTCGTGAAAGGAGC |
| *Cla013426* | ATTATGGTCGTCTTTCAGC | AGCCCTCTTTCACCTACA |
| *Cla019064* | CGAAATCAACGGCTACCA | CCAAACGGAATCACCTCA |
| *Cla001303* | TTTGTGTGTTCTTGTCCC | TCGTCATCGTCTGGTTTG |
| *Cla022253* | GAAGGGAACTATGGAACC | CACAAGGATGACAATGAAC |
| *GU565958* | ATTCTCCGTTTGGACCTTGCT | TCGTAGTTTTTCTCAATGGAGG |
| *Cla022180* | TCCACCAGGGTTTCGTCT | AAGCAACCTCGGACCTCA |
| *Cla012951* | GCCAACAAGGAGGATAGA | AGGAACAGTTTCAAGGGA |
| *Cla018703* | GGCTCTTGGAGTTGGAAA | TTCGTAGGCATCGACACG |
| *Cla022543* | CTTGTTCTTCCTCCACTTC | GTCTTGCCAGAGCATTAA |
| *Cla006520* | ACATCGGATCTAAGACAGC | CCAATTCCCTTCACAACT |
| *Cla005862* | AAGTGGATGATGGAGGTT | AGTGCCAGCAATGTAAGA |
| *Cla019322* | ATTTCTGCTGCTATGGTCG | CGTGTCGTATTGCCCTTC |
| *Cla018095* | CTTGGCTGGTTTGTTGGA | TTAGAGGTGGAGCAGAGTA |
| *Cla002910* | CTGACGGCTGAGGTTGTT | ACGGTGGAGATTGATTAGG |
| *Cla020261* | TGGTCGCAGGAGAAAGCA | CCACGAAGAGCCTCCACA |
| *Actin* | TGGGCTTTGCTCCTCTTAC | CCTTCGTGCTCATCTTACC |
| *Cla022180* | TCCACCAGGGTTTCGTCT | AAGCAACCTCGGACCTCA |
| *Cla012951* | GCCAACAAGGAGGATAGA | AGGAACAGTTTCAAGGGA |
| *Cla018703* | GGCTCTTGGAGTTGGAAA | TTCGTAGGCATCGACACG |
| *Cla022543* | CTTGTTCTTCCTCCACTTC | GTCTTGCCAGAGCATTAA |
| *Cla006520* | ACATCGGATCTAAGACAGC | CCAATTCCCTTCACAACT |
| *Cla005862* | AAGTGGATGATGGAGGTT | AGTGCCAGCAATGTAAGA |
| *Cla019322* | ATTTCTGCTGCTATGGTCG | CGTGTCGTATTGCCCTTC |
| *Cla018095* | CTTGGCTGGTTTGTTGGA | TTAGAGGTGGAGCAGAGTA |
| *Cla002910* | CTGACGGCTGAGGTTGTT | ACGGTGGAGATTGATTAGG |
| *Cla020261* | TGGTCGCAGGAGAAAGCA | CCACGAAGAGCCTCCACA |
